# Indoor Localization Algorithm Based on a High-Order Graph Neural Network

**DOI:** 10.3390/s23198221

**Published:** 2023-10-02

**Authors:** Xiaofei Kang, Xian Liang, Qiyue Liang

**Affiliations:** College of Communication and Information Engineering, Xi’an University of Science and Technology, Xi’an 710054, China; lx05356@163.com (X.L.); molly1242023@163.com (Q.L.)

**Keywords:** indoor localization, Wi-Fi fingerprint, adjacency matrix, graph neural network

## Abstract

Given that fingerprint localization methods can be effectively modeled as supervised learning problems, machine learning has been employed for indoor localization tasks based on fingerprint methods. However, it is often challenging for popular machine learning models to effectively capture the unstructured data features inherent in fingerprint data that are generated in diverse propagation environments. In this paper, we propose an indoor localization algorithm based on a high-order graph neural network (HoGNNLoc) to enhance the accuracy of indoor localization and improve localization stability in dynamic environments. The algorithm first designs an adjacency matrix based on the spatial relative locations of access points (APs) to obtain a graph structure; on this basis, a high-order graph neural network is constructed to extract and aggregate the features; finally, the designed fully connected network is used to achieve the regression prediction of the location of the target to be located. The experimental results on our self-built dataset show that the proposed algorithm achieves localization accuracy within 1.29 m at 80% of the cumulative distribution function (CDF) points. The improvements are 59.2%, 51.3%, 36.1%, and 22.7% compared to the K-nearest neighbors (KNN), deep neural network (DNN), simple graph convolutional network (SGC), and graph attention network (GAT). Moreover, even with a 30% reduction in fingerprint data, the proposed algorithm exhibits stable localization performance. On a public dataset, our proposed localization algorithm can also show better performance.

## 1. Introduction

Location-based services (LBSs) have been widely applied in various fields, providing services such as location identification and navigation to meet the diverse localization needs of different user groups [1]. In outdoor environments, mature global navigation satellite systems (GNSSs) can achieve satisfactory accuracy, fulfilling the requirements for most outdoor localization [2]. However, indoor localization still faces numerous challenges, including localization accuracy, implementation complexity, and the stability of localization in dynamically changing environments, due to the complexity of indoor environments [3].

With ongoing research on indoor localization technologies, numerous solutions have been proposed, including those based on Wi-Fi [4], Bluetooth [5], ultrasonic [6], vision [7], and so on. Among these, Wi-Fi-based indoor localization technologies have gained prominence in scenario applications due to the extensive deployment of Wi-Fi infrastructure and the widespread use of wearable smart devices [8,9]. Fingerprinting match and triangulation are two commonly used location estimation methods in indoor Wi-Fi localization technology. Triangulation localization methods generally have high localization accuracy. However, the accuracy depends heavily on precise prior knowledge of the channel model or meeting stringent clock synchronization requirements, and the performance drops significantly in non-line-of-sight (NLOS) environments [10]. In contrast, fingerprint-based localization methods have received widespread attention due to the estimation of location information through fingerprint matching [11]. This usually consists of two phases: offline and online. In the offline phase, a dataset is constructed with the locations in the localization area and their corresponding received signal strength (RSS). In the online phase, the location of the target point is predicted by matching the real-time RSS received by the mobile device with the information stored in the dataset.

Considering that fingerprint localization methods can essentially be modeled as supervised learning problems, several traditional machine learning algorithms have been successfully applied to solve the challenges in fingerprint localization [12]. To further improve the accuracy of localization, deep learning (DL)-based models have also been used to improve the performance of fingerprint localization. These deep learning localization models, such as deep neural networks (DNNs) [13], convolutional neural networks (CNNs) [14], and autoencoders (AEs) [15], have been designed to adapt to the structured nature of spatially distributed data [16]. However, in complex and dynamically changing indoor environments, the amount of RSS received can be time-varying, giving fingerprint datasets an unstructured nature. These models often struggle to effectively capture the unstructured data characteristics of Wi-Fi fingerprints in different propagation environments. In addition, they often ignore the spatial relationships between the relative locations of access points (APs) that transmit signals in indoor environments. The complexity of such unstructured data poses a huge challenge for popular machine learning algorithms.

The graph neural network (GNN) [17] provides a novel solution for feature extraction and data processing of unstructured data, making it a standard approach for handling graph-based data. Chiou [18] proposed a graph-neural-network-structure-based indoor localization method using multi-view images. In this graph construction method, the front and back views were only modeled as second-order neighbors, which could potentially result in a loss of some relational information. Lezama [19] proposed a graph localization method that solely relied on RSS and did not utilize indoor floor plans. Zhou [20] designed a graph-based semi-supervised method that needed to compute the distance between each pair of fingerprints. This computation grew exponentially when dealing with large fingerprint datasets.

Based on the above analysis, in order to utilize the unstructured data information in Wi-Fi fingerprint data and mine the spatial features between the relative locations of Aps, in this paper, we propose an indoor localization algorithm based on a high-order graph neural network (HoGNNLoc). Firstly, we design a preprocessing step to transform fingerprint data with unstructured data features into a graph representation based on relative AP locations. Based on this graph structure, a high-order graph neural network is constructed to extract and aggregate features. Finally, the fully connected network utilizes fingerprint representation for location estimation. The algorithm can effectively reduce the localization error and improve the stability of location prediction to meet the requirements of accurate localization in complex and dynamically changing indoor environments.

The rest of this paper is organized as follows: Section 2 introduces the principle of the proposed algorithm, Section 3 explains the mathematical model, the experimental results and analysis are presented in Section 4, and, finally, Section 5 concludes the paper.

## 2. Algorithm Localization Principle

Wi-Fi based indoor localization aims to accurately estimate a user’s location information in complex indoor environments that consist of multiple APs and reference points (RPs). Due to the diverse propagation environments, the generated data exhibit non-Euclidean structures. To address this, a GNN can be applied by constructing graph feature representations to extract the inherent spatial characteristics of the data structure.

**Figure 1:**
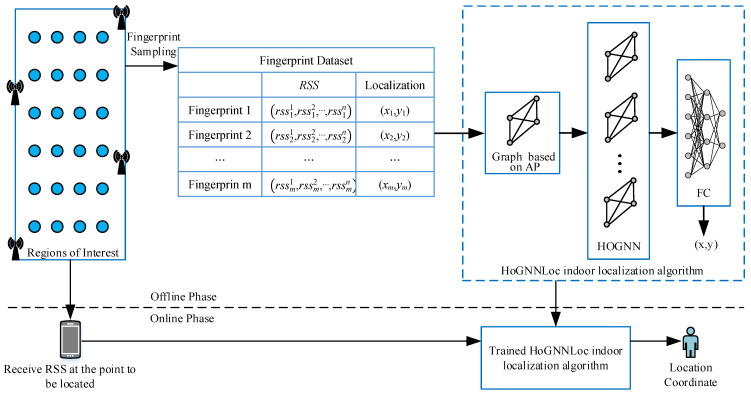
Indoor localization method based on a high-order graph neural network.

The structure of our method is shown in Figure 1, which can be divided into two phases: offline and online. In the offline phase, the RSS value and location coordinate information of each RP are first collected to construct a location fingerprint dataset. The sample data of this fingerprint dataset consist of a set of RSS values collected from specific RP coordinates. Correspondingly, the labels of samples are the coordinates of this RP. It is crucial to establish a complete and effective graph-based representation to capture the key features of the data structure for the application of a high-order graph neural network (HoGNN). To explore the topological structure of the fingerprint data and exploit the spatial characteristics of APs, each AP is considered as a node in the constructed graph structure. The design of an adjacency matrix represents the edge connection relationships in the graph structure based on the location relationships between APs. The RSS received at the RP is treated as the nodes’ input feature, and the node feature is aggregated and updated. After that, the feature vectors are mapped to the location space through the fully connected network, resulting in the precise localization of the predicted RP location coordinates $\left(x,y\right)$. In the online phase, the point to be located will input the received RSS value into the trained HoGNNLoc and output the predicted location of the point to be located, thus completing the localization process.

This process requires insight into the complexity involved in mapping the relationships between data points. Therefore, a graph structure is constructed to depict possible unstructured data relationships, enabling the utilization of HoGNN to unlock the potential of Wi-Fi-fingerprint-based localization technology.

## 3. Algorithm Localization Model

### 3.1. Problem Formulation

Considering an indoor environment with *N* APs and *M* RPs, the fingerprint dataset consists of the locations of RPs and their corresponding RSS values. The location coordinates of RPs can be stored as $𝒫=\left\{p_{1},p_{2},\ldots ,p_{M}\right\}$, where $p_{m}=\left[x_{m},y_{m}\right]$ represents the location coordinates of RP*_m_*. The location fingerprint dataset can be denoted as $R=\left\{r_{1},r_{2},\ldots ,r_{M}\right\}$, where $r_{m}=\left[rss_{m}^{1},rss_{m}^{2},\ldots ,rss_{m}^{n}\right]$ indicates the set of RSS vectors received at RP*_m_*, and $rss_{m}^{n}$ indicates the signal strength of the nth AP received at RP*_m_*. Given $r_{m}\in R$ as a set of signal strength values and its location coordinate $p_{m}\in 𝒫$, our goal is to learn a function F:R→𝒫 that maps the input $r_{m}$ to the location coordinate $p_{m}$.

### 3.2. Graph Structure Based on Adjacency Matrix Design

**Figure 2:**
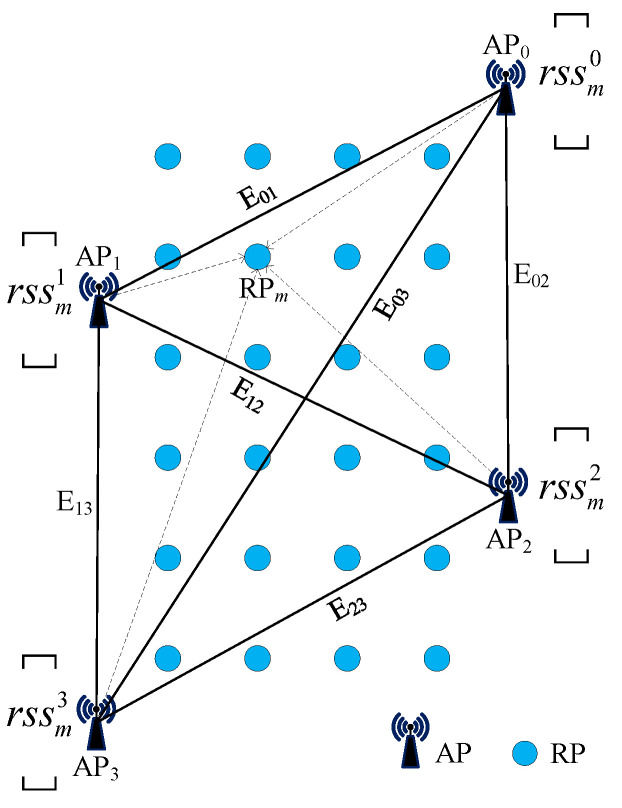
Construction of the graph structure.

To capture the interrelationships among APs in an indoor environment and achieve reliable location prediction by effectively handling the unstructured data of the fingerprints, the model needs to leverage and combine the features of neighboring nodes. Therefore, we construct a fully connected undirected graph $G=\left(V,E\right)$ with each AP as a node based on the relative locations among the APs. In this graph, each node $V_{n}\left(n\in \left\{0,1,\ldots ,N\right\}\right)$ represents an actual AP in the environment, and each edge $E_{ij}\left(i,j\in \left\{0,1,\ldots ,N\right\}\right)$ represents the adjacency relationship between node $v_{i}$ and node $v_{j}$. The initial feature of each node is the signal strength $rss_{m}^{n}$ received from the nth AP at ${\mathrm{RP}}_{m}$. The graph structure is constructed as shown in Figure 2. The performance gains from graph data are closely related to the number and quality of adjacent nodes. During the localization process, the deployed APs in the indoor environment, due to their different distances from the target location, may provide positive information or introduce negative interference into the localization results. Therefore, in the design of the adjacency matrix A, each edge is assigned a weight corresponding to the reciprocal of the Euclidean distance between the two neighboring nodes. The construction of the adjacency matrix follows the following principles:

(1) $$A_{i,j}=A_{j,i}=\left\{\begin{array}{l} 0\;\;\;\;\;\;i=j\\ \frac{1}{d_{ij}}\;\;\;\;i\neq j \end{array}\right.$$

where $d_{ij}$ denotes the Euclidean distance between node $v_{i}$ and node $v_{j}$.

### 3.3. Network Model

The GNN utilizes aggregators to gather neighborhood information [21]. Examples of aggregators include mean aggregators, which take the average of neighboring feature vectors, sum aggregators, which apply summation, and attention aggregators, which take the weighted sum. By aggregating representations of neighbors and iteratively updating their representations, the hidden feature states h of nodes can be updated using a basic GNN model:

(2) $$h^{\left(t\right)}\left(v\right)=\sigma \left(h^{\left(t-1\right)}\left(v\right)\cdot W_{1}^{\left(t\right)}+\sum h^{\left(t-1\right)}\left(u\right)\cdot W_{2}^{\left(t\right)}\right)$$

where v and u represent the nodes with adjacency in the graph structure, $W_{1}^{\left(t\right)}$ and $W_{2}^{\left(t\right)}$ are the parameters to be learned, $\sigma \left(\cdot \right)$ represents the activation function, and $h^{\left(t\right)}\left(\cdot \right)$ is the hidden state information of the node in the iteration. When considering a node, it establishes correspondences with its neighboring nodes and obtains a new feature vector. The node also incorporates information from its own previous state.

By associating GNN operations with the Weisfeiler–Leman (WL) graph isomorphism heuristic, it has been shown that the GNN exhibits the same expressive power as that of 1-WL in distinguishing non-isomorphic graphs [22]. Inspired further by k-WL, the HoGNN demonstrates the great potential of learning a graph structure by efficiently using higher-order information to acquire structural features, making the information update process occur between subsets of nodes. The HoGNN is applied to the graph G constructed above for message passing, $r_{m}$ received by the point to be located is taken as input, the hidden state $h_{n}$ of the node is aggregated and updated, the internal interactions and the interactive information between features are explored, and Wi-Fi regression mapping between fingerprint data and location coordinates is learned.

Assuming that the set of all nodes in graph G can be represented as $\left[V\left(G\right)\right]$, let $\left[V{\left(G\right)}^{k}\right]$ be a subgraph of $\left[V\left(G\right)\right]$, and let $V=\left\{v_{1},\ldots ,v_{k}\right\}$ be a set of k nodes in $\left[V{\left(G\right)}^{k}\right]$. The neighborhood of v is defined as follows:

(3) $$𝒩\left(v\right)=\left\{t\in {\left[V\left(G\right)\right]}^{k}\left|\right.\;\left|v\cap t\right|=k-1\right\}$$

Equation (3) means that a neighbor of subset v is another subset t with k−1 nodes. A local neighbor ${𝒩}_{L}\left(v\right)$ of v consists of all elements in the set $t\in 𝒩\left(v\right)$. The computational procedure for message passing of hidden features between v and its local neighbors is

(4) $$h_{k,L}^{\left(t\right)}\left(v\right)=\sigma \left(h_{k,L}^{\left(t-1\right)}\left(v\right)\cdot W_{1}^{\left(t\right)}+\sum_{u\in {𝒩}_{L}\left(v\right)}h_{k,L}^{\left(t-1\right)}\left(u\right)\cdot W_{2}^{\left(t\right)}\right)$$

where $h_{k,\;L}^{\left(t-1\right)}\left(v\right)$ represents the feature vector of node set v located at layer t−1, and the aggregation of local neighbor features of v represented by u is $\sum_{u\in {𝒩}_{L}\left(v\right)}h_{k,L}^{\left(t-1\right)}\left(u\right)$. The message-passing process represented by Equation (4) can also be expressed as

(5) $${\hat{h}}_{i}=W_{1}h_{i}+W_{2}\sum_{j\in 𝒩\left(i\right)}e_{j,i}\cdot h_{j}$$

where $W_{1}$ and $W_{2}$ are the parameters to be learned, $h_{i}$ represents the feature vector of node set i, $\sum j\in 𝒩\left(i\right)$ represents the set of local neighbors of i represented by j, the weight of local neighbor edges is represented by $e_{j,i}$, and $h_{j}$ represents the feature vector of local neighbors. The ReLU activation function is also used in the message-passing process expressed in Equation (5).

After the message passing, the final hidden feature vectors are aggregated to form a single graph feature representation ${\hat{r}}_{m}=\left[{\hat{h}}_{1},{\hat{h}}_{2},\ldots ,{\hat{h}}_{n}\right]$, which is then passed to a fully connected (FC) network, which maps the connected feature vectors to the two-dimensional coordinates of the location space:

(6) $${\hat{p}}_{m}=f\left(\mathrm{FC}\left({\hat{r}}_{m}\right)\right)$$

Using the mean square error (MSE) as the loss function, the final output is used for the indoor localization regression task:

(7) $$L=\frac{1}{M}\left(\sum_{m=1}^{M}{\left(p_{m}-{\hat{p}}_{m}\right)}^{2}\right)$$

where $p_{m}=\left[x_{m},y_{m}\right]$ represents the actual location coordinates of the input $r_{m}$, and ${\hat{p}}_{m}=\left[{\hat{x}}_{m},{\hat{y}}_{m}\right]$ represents the coordinates of the model prediction output.

## 4. Experiments 

### 4.1. Fingerprint Dataset

#### 4.1.1. Self-Built Underground Garage Dataset

**Figure 3:**
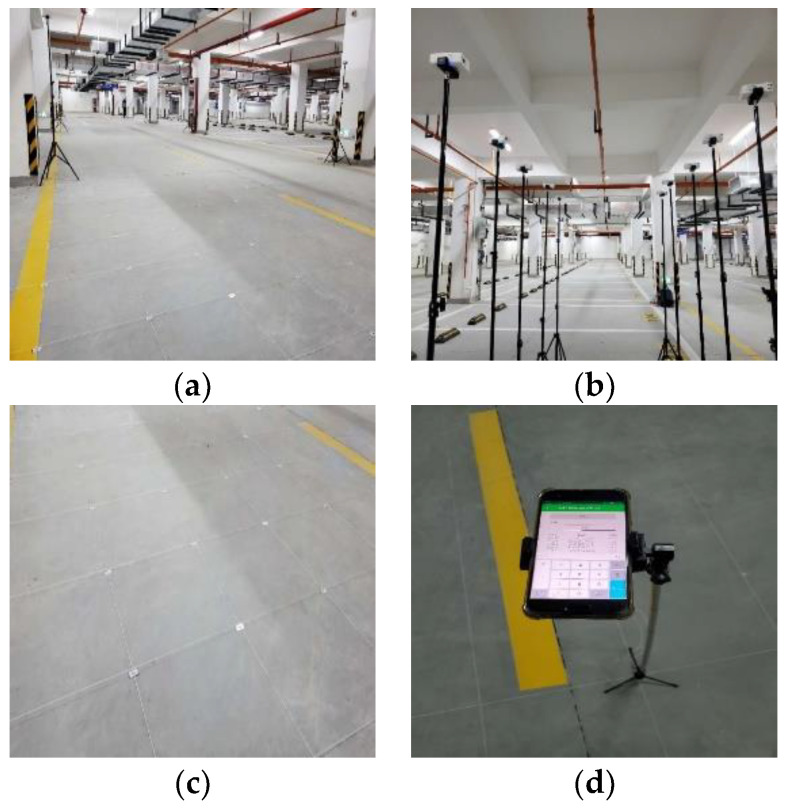
Experimental environment; (**a**) experimental site; (**b**) AP equipment; (**c**) grid division; (**d**) receiving equipment.

We chose an area of 40 m × 5.5 m in an underground garage as the experimental site, and its layout is shown in Figure 3a. Ten APs with the same model and a height of 2 m were placed in the area, as shown in Figure 3b; the coordinates of the APs on the plane were (0.0, 7), (5.5, 7), (0.0, 15), (5.5, 15), (0.0, 23), (5.5, 23), (0.0, 31), (5.5, 31), (0.0, 39), and (5.5, 39). The area was divided into small grids of 0.5 m × 0.5 m, as shown in c; each vertex of these grids was assigned as an RP, resulting in a total of 972 RPs. The establishment of the offline fingerprint dataset was realized through mobile phone application software that we developed, and the RSS value of each AP received by each RP point was tested and recorded, as shown in Figure 3d. Then, 80% of the collected fingerprint data were divided into training data, and the remaining 20% of the data samples were used for testing. 

**Figure 4:**
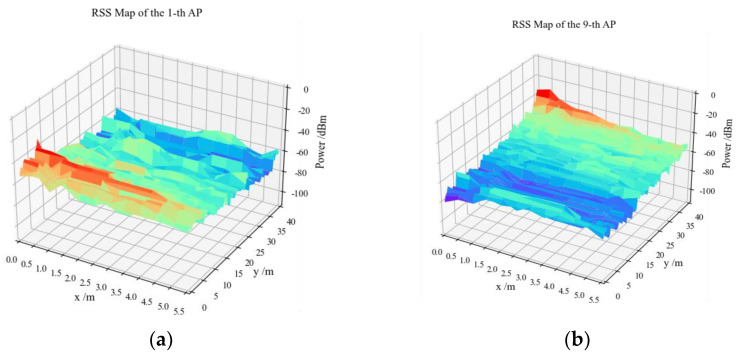
Visualization of the RSS values: (**a**) the first AP; (**b**) the ninth AP.

The spatial distributions of the RSS of the first and ninth APs in the fingerprint dataset are shown in Figure 4, where the red peak indicates the locations of APs.

#### 4.1.2. SoLoc Dataset

The SoLoc dataset [23] was established within a laboratory measuring 38 m × 50 m in its dimensions. In this context, 128 fingerprint samples were designated for training, with the remaining 475 data samples being allocated for testing. A feature distinguishing this dataset from our dataset was that the training set for this dataset contained a much smaller number of fingerprints than the test dataset. Additionally, it is worth highlighting that this dataset was created in a relatively spacious indoor environment.

### 4.2. Algorithm Model Configuration

In the graph structure, an undirected edge was implemented with two directed edges in opposite directions. In the model structure, the dimension of the hidden state ${\hat{h}}_{i}$ was 20. The fully connected network output dimension was 2, representing the two-dimensional coordinates of the predicted location, as shown in Table 1. The randomness of the original structure was increased by adding a Dropout layer to randomly invalidate some neuron nodes, thus stabilizing the training by determining the outcome of multiple random structures. The algorithm model was implemented based on Pytorch and was trained in an end-to-end manner; the main parameters are shown in Table 2.

Based on the above fingerprint datasets, we compared the localization performance of the proposed HoGNNLoc localization algorithm with those of K-nearest neighbors (KNN), a DNN, a simple graph convolutional network (SGC) [24], and a graph attention network (GAT) [25]. A comparison is made to verify the superiority of the proposed localization algorithm. The model structure of the first two graph neural network algorithms was the same as the model structure of the proposed algorithm. The DNN consisted of three layers of fully connected layers, and the dimension of the hidden layer was 128.

### 4.3. Results and Analysis

#### 4.3.1. Results on the Self-Built Underground Garage Dataset

**Figure 5:**
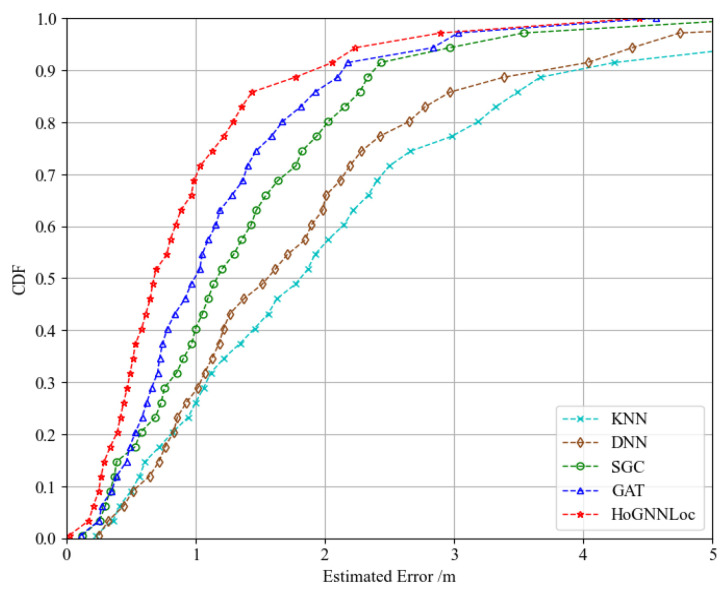
CDF of performance comparison of different localization algorithms.

Figure 5 illustrates a performance comparison of the proposed HoGNNLoc localization algorithm with different existing localization algorithms using the cumulative distribution function (CDF) of errors. The experimental results reveal noticeable differences in the localization outcomes obtained with the different localization algorithms. The localization error of HoGNNLoc was consistently smaller than that of the other localization algorithms under equal probability conditions. At the 80th percentile, its localization accuracy remained within 1.29 m. Compared with the localization accuracies of KNN (3.18 m), the DNN (2.65 m), the SGC (2.02 m), and the GAT (1.67 m), the improvements were 59.2%, 51.3%, 36.1%, and 22.7%.

**Figure 6:**
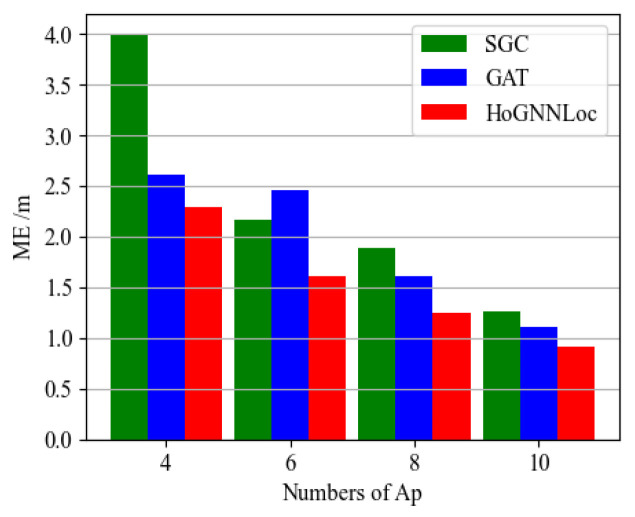
ME comparison of the neural network localization algorithms with different AP numbers.

To analyze the impact of the number of APs on the localization results, we conducted experiments on the fingerprint data aggregation process to investigate the influence of selecting different quantities of APs on the localization algorithm. Figure 6 shows the mean error (ME) of each localization algorithm when using 4, 6, 8, and 10 APs. The results indicated that the localization outcomes of the various graph-neural-network-based algorithms were affected by the selection of the AP quantity. Furthermore, across different AP quantity selections, HoGNNLoc consistently outperformed the other two graph-neural-network-based localization algorithms in terms of the average localization accuracy.

**Figure 7:**
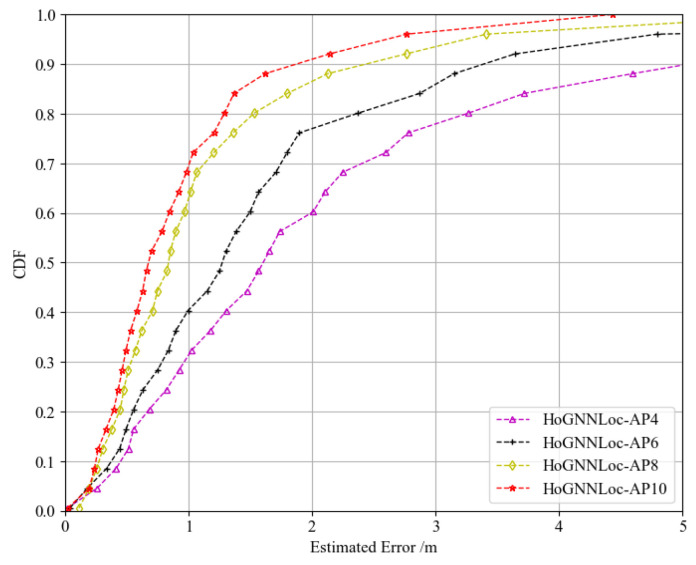
CDF of the comparison of the performance of HoGNNLoc with different numbers of APs.

Comparing the average localization error only provided a macro-level assessment of the performance of the localization algorithms, making it difficult to conduct a detailed analysis of their localization effects. In order to analyze the impact of AP quantity on the localization performance of the proposed algorithm more comprehensively, we generated localization CDF plots specifically for HoGNNLoc with 4, 6, 8, and 10 APs, as shown in Figure 7. The results demonstrated that as the number of APs increased, the localization performance of HoGNNLoc improved. This indicated that increasing the number of APs not only provided an ample amount of data required by the algorithm, but also enhanced the informative characteristics of the data, thereby improving the localization performance of the algorithm.

**Figure 8:**
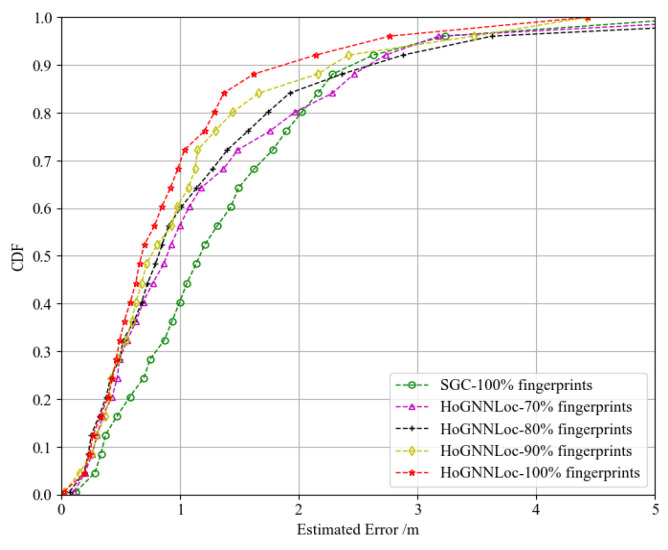
CDF of the performance comparison with different numbers of fingerprint samples.

We conducted experiments using different quantities of fingerprint data samples (70%, 80%, 90%, and 100%) to evaluate the stability of the proposed HoGNNLoc algorithm’s localization performance in dynamic environments. As shown in Figure 8, as the quantity of fingerprint data decreased, the algorithm demonstrated good stability in terms of localization performance. The graph features captured prior geometric information from the APs, enhancing the algorithm’s generalization capability. Even with a 30% loss of data samples, the overall localization performance of the HoGNNLoc algorithm remained superior to that of the SGC algorithm, which used 100% of the samples.

**Figure 9:**
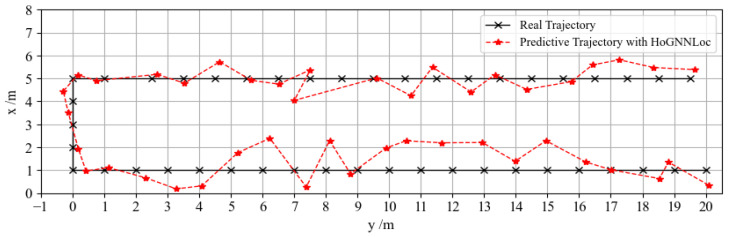
Localization path trajectory comparison.

Furthermore, to evaluate the tracking performance of the proposed HoGNNLoc algorithm in dynamic environments, we selected a specific path within the experimental scenario. Figure 9 shows a comparison of the path trajectories, where the actual path is depicted as a black dashed curve, and the path trajectory predicted by HoGNNLoc is represented by a red dotted curve. The results depicted in the figure demonstrate a striking similarity between the overall localization trajectory and the actual path, indicating high localization accuracy and robust tracking performance.

The response time is also a crucial metric for evaluating indoor localization performance. After running 1000 epochs, we recorded the average training time per epoch and the average testing time per test for the various deep learning localization algorithms, as shown in Table 3. Our proposed HoGNNLoc localization algorithm outperformed the other graph-neural-network-based localization algorithms in both training and testing times. While the DNN-based localization algorithms exhibited faster training and testing times, their localization performance failed to meet our specific localization requirements. These differences in test times are still acceptable as far as indoor localization accuracy is concerned.

#### 4.3.2. Results Based on the SoLoc Dataset

**Figure 10:**
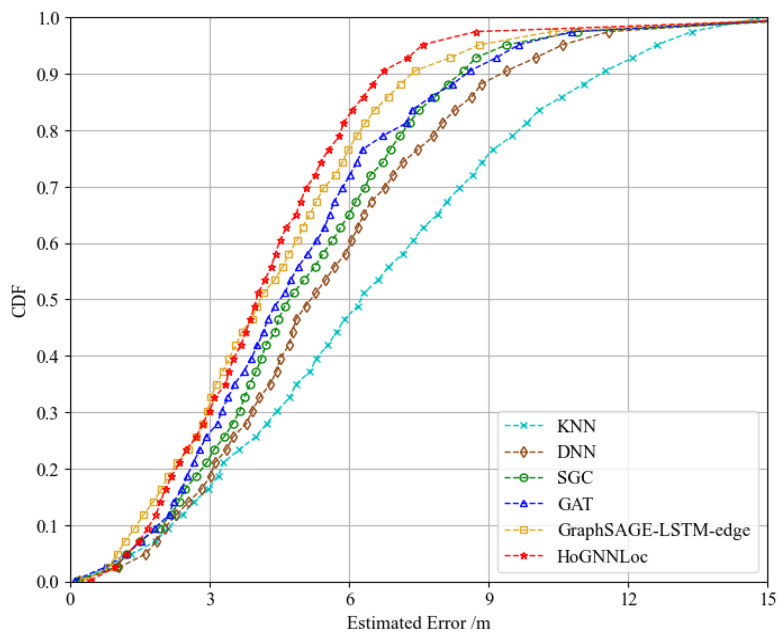
CDF of the performance comparison of different localization algorithms.

To further validate the performance of the proposed localization algorithm, we conducted experiments on the publicly available SoLoc dataset, specifically focusing on scenarios using a single Wi-Fi signal source. We compared the performance of our algorithm with the localization methods outlined in [26]. The plotted error CDF gave similar performance results to those on the self-built underground garage dataset, as shown in Figure 10. Our proposed HoGNNLoc localization algorithm offered superior localization accuracy. At the 80th percentile, its localization accuracy remained within 5.81 m. Compared with the localization accuracies of KNN (9.62 m), the DNN (7.92 m), the SGC (7.21 m), the GAT (7.03 m), and GraphSAGE-LSTM-edge (6.25 m), the improvements were 39.6%, 26.6%, 19.4%, 17.4%, and 7.04%.

Furthermore, we calculated the lower quartile, median, and upper quartile of the mean absolute error to assess the stability of different localization algorithms, as presented in Table 4. From the experimental results, it is evident that our proposed HoGNNLoc localization algorithm exhibited better stability in location prediction.

## 5. Conclusions

In this paper, we propose an indoor localization algorithm based on a high-order graph neural network (HoGNNLoc). The localization task incorporates basic geometric information about APs and builds a graph structure based on its a priori location information. This graph structure is used to construct a higher-order graph neural network that can fully explore the unstructured features present in the Wi-Fi fingerprint data and learn valid structural information. Finally, localization is achieved by using the designed fully connected network. The algorithm can effectively improve localization accuracy and has strong stability, which is well suited to meet the needs of accurate localization in complex and dynamically changing indoor environments.

## Figures and Tables

**Table 1:** The model’s structural parameters.

Network Layer	Input Shape	Output Shape	Activation	Dropout
HoGNN	1 × *N*	20 × *N*	ReLU	Yes
FC1	20 × *N*	64	ReLU	No
FC2	64	2	No	No

**Table 2:** Parameters in the experiment.

Parameter Name	Parameter Value
Dropout	0.3
Activation	ReLU
Optimizer	Adam
Learning Rate	0.0005
Loss Function Epoch	MSE 1000
Batch Size	32

**Table 3:** Response times of the localization algorithms.

Localization Algorithms	Average Offline Training Time per Epoch (ms)	Average Online Testing Time per Time (ms)
DNN	102.67	0.06
SGC	133.75	0.10
GAT	189.24	0.11
HoGNNLoc	126.16	0.07

**Table 4:** Error data distribution for the localization algorithms.

Localization Algorithms	Lower Quartile (m)	Median (m)	Upper Quartile (m)
KNN	3.85	5.98	7.97
DNN	3.46	5.17	7.22
SGC	3.09	4.64	7.10
GAT	2.77	4.33	6.60
GraphSAGE-LSTM-edge	2.28	3.52	5.13
HoGNNLoc	2.26	3.24	5.32

## Data Availability

Not applicable.
